# Cytokinin-Based Tissue Cultures for Stable Medicinal Plant Production: Regeneration and Phytochemical Profiling of *Salvia bulleyana* Shoots

**DOI:** 10.3390/biom11101513

**Published:** 2021-10-14

**Authors:** Izabela Grzegorczyk-Karolak, Katarzyna Hnatuszko-Konka, Marta Krzemińska, Monika A. Olszewska, Aleksandra Owczarek

**Affiliations:** 1 Department of Biology and Pharmaceutical Botany, Medical University of Lodz, 90-151 Lodz, Poland; marta.wojciechowska2@stud.umed.lodz.pl; 2 Department of Molecular Biotechnology and Genetics, Faculty of Biology and Environmental Protection, University of Lodz, 90-237 Lodz, Poland; katarzyna.hnatuszko@biol.uni.lodz.pl; 3 Department of Pharmacognosy, Medical University of Lodz, 90-151 Lodz, Poland; monika.olszewska@umed.lodz.pl (M.A.O.); aleksandra.owczarek@umed.lodz.pl (A.O.)

**Keywords:** auxin, callus, cytokinins, direct and indirect organogenesis, genetic stability, ISSR analysis, polyphenolic compounds, rosmarinic acid

## Abstract

*Salvia bulleyana* is a rare Chinese medicinal plant that due to the presence of polyphenols lowers the risk of some chronic diseases especially those related to the cardiovascular system. The present study examines the organogenic competence of various combinations and concentrations of plant growth regulators to develop an efficient protocol for in vitro regeneration of *S. bulleyana* via leaf explants, maintaining the high production of active constituents. The purpose of the study was also to assess the possibilities of using a cytokinin-based regeneration to effectively produce therapeutic compounds. The adventitious shoot formation was observed through direct organogenesis on media with purine derivatives (meta-topolin, mT and benzylaminopurine, BAP), and through indirect organogenesis on media with urea derivatives (tidiazuron, TDZ and forchlorfenuron, CPPU). The highest regeneration frequency (95%) with 5.2 shoots per explant was obtained on leaves cultured on Murashige and Skoog (MS) medium containing 0.1 mg/L naphthalene-1-acetic acid (NAA) and 2 mg/L BAP. Following inter simple sequence repeat (ISSR) marker-based profiling, the obtained organogenic shoot lines revealed a similar banding pattern to the mother line, with total variability of 4.2–13.7%, indicating high level of genetic stability. The similar genetic profile of the studied lines translated into similar growth parameters. Moreover, HPLC analysis revealed no qualitative differences in the profile of bioactive metabolites; also, the total polyphenol content was similar for different lines, with the exception of the shoots obtained in the presence of CPPU that produced higher level of bioactive compounds. This is the first report of an effective and rapid in vitro organogenesis protocol for *S. bulleyana*, which can be efficiently employed for obtaining stable cultures rich in bioactive metabolites.

## 1. Introduction

Plant tissue culture (PTC) is unquestionably a powerful tool that can be used to continuously reproduce commercially-important crops [1]. In vitro culture allows fast and efficient multiplication of disease-free, sometimes endangered, plant species. With the increasing reliance on plant-derived compounds and materials, the value of in vitro culture has grown exponentially. In addition to foods, crops are sources of a range of non-food goods, such as nutrients, fiber, dyes, pharmaceuticals, insecticides or cosmetics; as such, there is a need for more efficient strategies of boosting their production while conserving their natural pool [1]. Even when metabolic engineering is valuable and gives new opportunities, the regeneration is still the crucial factor that ensures the efficacy and profitability of the process. Moreover, successful acquisition of plant resources in vitro requires a detailed understanding of the biochemical and phytochemical characteristics of potential raw materials. Hence, there is a need for studies on available methods of tissue culture. In addition, an effective regeneration protocol has to incorporate a simple medium formulation, minimum production stages, reproducible level of propagation and genetically stable products.

Plants typically start the process of regeneration via one of two different cellular strategies, which include either programming of differentiated somatic cells or reactivation of relatively undifferentiated cells. Regeneration depends on cellular plasticity, which can be defined as the ability to re-specify the cell fate [2,3]. Although, the totipotency of plant cells theoretically allows them to be used as the base for regenerating an entire new plant, this process is influenced by genotype, explant age and differentiation level, among others [4,5]. Above all, however, the plant plasticity is directed by phytohormones used in media. It is common knowledge, that plant hormones are capable of affecting many vital processes, comprising plant growth and development, environmental adaptation and response to biotic and abiotic stressors. All of this makes phytohormones a tool of choice for biotechnological manipulation providing plants with high regeneration and wide application. This manipulation includes also a hormone-induced desired changes in phytochemical profile, resulting in the increase of therapeutic metabolite production. However, while a lot of effort has been employed to design culture protocols for model and cultivated plants, it is worth noting that some of potentially commercial species still lack the effective in vitro techniques for promoting biomass propagation. This is also the case of therapeutically valuable *Salvia bulleyana*.

*S. bulleyana* is a member of the genus Salvia, well-known for its therapeutic potential. It is used in Chinese medicine as Danshen, an agent used in coronary heart disease for the alleviation of angina pectoris, coronary artery spasm and myocardial infarction [6,7]. It has been also used as a tranquilizer and to resolve swelling. *S. bulleyana* is known to be a rich source of polyphenolic acid and a promising medical bioresource with 28 phenolic compounds being detected in hydromethanolic extracts from shoots and roots of the species [8,9]. Among polyphenolic acids or their derivatives, the main secondary metabolite is rosmarinic acid in the shoots, and salvianolic acid K in the roots. In addition to polyphenolic acids, flavone glycosides were also detected in the shoot extract.

Its promising medicinal potential and rarity unquestionably place *S. bulleyana* among the candidates for the establishment of an in vitro regeneration method. Since members of the genus *Salvia* generally display strong apical dominance, the question of an alternative effective propagation method arises for *S. bulleyana*. Hence, the cytokinin-induced organogenesis may become a tool of choice to increase bud regeneration rate. Therefore, this study aimed to propose an organogenic pathway of *S. bulleyana* as a reliable strategy of propagation. The first part of the investigation included optimization of shoot organogenesis with an assessment of regeneration rate. For this purpose, a set of cytokinins triggering the organogenesis pathway was tested. Moreover, as cryptic genetic defects arising via in vitro propagation may seriously limit the utility of micropropagation system, the regenerated shoots were analyzed for possible somaclonal variation using ISSR (inter simple sequence repeat). The study was also accompanied by phytochemical analysis of the obtained cultures to determine the effect of the applied cytokinin-based regenerative methods on the production of bioactive compounds.

## 2. Materials and Methods

### 2.1. Plant Material

*S. bulleyana* seeds were derived from the botanical garden of the University of Bonn (Bonn, Germany). They were surface sterilized with 5% sodium hypochlorite for 10 min, and rinsed three times with sterile distilled water. Surface-sterilized seeds were placed on agar (0.7% *w/v*) Murashige and Skoog (MS) medium [10] containing 3% (*w/v*) sucrose, 0.02 mg/L kinetin and 1 mg/L gibberellic acid and germinated in the growth chamber at 26 ± 2 °C in the dark. The pH of the medium was adjusted to 5.8 using NaOH or HCl before autoclaving at 121 °C and 103 kPa for 20 min. The shoot tips of 4-week-old seedlings were used to initiate the original shoot culture and were transferred on MS agar medium with 0.1 mg/L IAA (indoliloacetic acid) and 1 mg/L BAP (benzylaminopurine). The culture was kept at 26 ± 2 °C under a 16 h photoperiod provided by cool white fluorescent lamps (40 µM/m s) and subcultured every 5 weeks. This culture served as a source of extracts for organogenesis and was a clonally propagated (from the existing meristems) a control culture.

### 2.2. Optimization of Shoot Regeneration

Leaves from the 5-week-old in vitro cultivated shoots were used to optimize shoot organogenesis. They were used to regenerate shoots on MS medium supplemented with 0.1 or 0.5 mg/L NAA (naphthalene-1-acetic acid) and different cytokinins: benzyloaminopurine (BAP), meta-topoline (mT), tidiazuron (TDZ) or forchlorfenuron (CPPU) at different concentration (0.2, 0.5 and 1 mg/L for TDZ, and 1, 2, 4 mg/L for others) and solidified with 0.7% agar. All the cultures were maintained in a growth chamber at 26 ± 2 °C in a 16 h photoperiod and 8 h dark cycles with 40 µM/m∙s of light intensity.

In order to assess the influence of different PGRs on shoot proliferation after 5 weeks, the following growth parameters were measured: callus regeneration frequency (%), callus morphology, adventitious bud regeneration frequency (%), mean number of shoots/buds on explant.

### 2.3. In Vitro Propagation

The shoots obtained from the various conditions were transferred to MS medium with 0.1 mg/L and 1 mg/L BAP and cultivated under conditions as described above for organogenesis (temp. 26 ± 2 °C, 16 h photoperiod, subcultured every 5 weeks). Finally, four shoot lines were selected and cultivated, each obtained in medium with a different cytokinin. All of these cultures, as well as the control culture, were run under the same conditions and subcultured every 5 weeks. After the cultures were stabilized (passages 9–11), their growth parameters were assessed and compared. For this purpose, at the end of the five-week culture, the percentages of explants that produced buds, the mean number of buds/shoots per explant, main and adventitious shoot length and culture biomass (fresh and dry weight per tube) were recorded.

### 2.4. Genetic Analysis

#### 2.4.1. DNA Extraction

Genomic DNA was extracted from the regenerated shoots following the CTAB protocol [11]. The plant tissue was weighed to obtain 100 mg of each sample, then the material was ground in liquid nitrogen in an Eppendorf microtube and subjected to proper preparation. The quality of the isolated DNA was evaluated by 0.7% agarose gel electrophoresis (Bio-Rad, Warsaw, Poland). The purity of DNA samples was then assessed by the spectrometric estimation of the A260/A280 ratio (with purity coefficient ranged from 1.84 to 1.92).

#### 2.4.2. ISSR-PCR

The extracted DNA samples were submitted to ISSR-PCR amplification. Based on existing literature, 15 primers (13 representing UBC primers set collected in University of British Columbia, Vancouver, Canada) were selected to investigate the genetic stability of regenerated shoots (Table 1) (Erbano et al. 2015). PCR reactions were accomplished in a total 20 µL reaction volume. The sample contained 10 mL master mix 2XPCR (ready-to-use Color OptiTaq PCR Master Mix 2x) (EURx^®^ Molecular Biology Products, Gdansk, Poland), 0.5 µL of plant genomic template (≈50 ng DNA) and 9.5 µL of ddH_2_O. Amplification was carried out in an Termocycler Biometra UNO II under the following reaction conditions: 94 °C—2 min (initial denaturation) followed by 35 cycles of 94 °C—1 min, adequate annealing temperature (presented in Table 1) for 45 s, 72 °C—2 min and a finishing extension at 72 °C—7 min. The regular reaction mix without template plant DNA was used as the negative control. The amplification products were split by electrophoresis in 2% agarose gel in 1 × TAE (Tris-acetate-EDTA) buffer and stained with ethidium bromide (Merck, Darmstadt, Germany). The size of the obtained fragments was estimated using a FastGene 100 bp DNA Marker (NIPPON Genetics, Düren, Germany), covering length ranging from 100 to 3000 bp.

#### 2.4.3. Molecular Data Analysis Regeneration and Proliferation

ISSR reactions were performed three times and the reproducible amplification products were scored. The banding profiles of the unambiguous and reproducible bands generated by ISSR primers were scored. The data were pooled into a binary matrix based on the presence (+) or absence (−) of the selected bands. Jaccard’s coefficient was used to estimate genetic similarity of the regenerated cultures [12]. The value of the coefficient lies between 0 and 1 (0 indicates no set similarity, while 1 is complete overlapping).

### 2.5. Identification and Accumulation of Polyphenolic Compound

Lyophilized plant material (100 mg) from four different culture lines and controls, after 10 passages (*cir*. one year) in in vitro conditions, was pulverized and extracted with methanol: water (4:1, *v/v*) using a UD ultrasonic disintegrator (Techpan, Warsaw, Poland) as described previously [8]. The obtained extracts were evaporated under reduced pressure and stored in a refrigerator at 2–4 °C before analysis. The metabolite profile of the hydromethanolic extracts was determined using UPLC-DAD/ESI-MS as presented previously [8]. An analysis was performed using a UPLC-3000 RS system (Dionex, Germering Germany) equipped with an AmaZon SL ion trap mass spectrometer with an ESI interface (Bruker Daltonik GmbH, Bremen, Germany) and a PDF detector, and a Zorbax SB-C18 column (150 mm × 2.1 mm, 1.9 µm; Agilent, Santa Clara, CA, USA). The compounds were identified on the basis of MS spectra, UV absorption spectra and retention time according previous data. The quantitative analysis was applied with an Elite LaChrom Hitachi system and a Ascentis^®^ C18 Superguard guard column (3 µm × 20 mm, 4 mm) as described by Wojciechowska et al. [9]. Standard calibration curves were constructed based on the peak area. Reference sample of caffeic acid were purchased from Sigma Aldrich/Merck (Darmstadt, Germany), rosmarinic acid from Extrasynthese (Genay, France), and salvianolic acid A and salvianolic acid F from ChemFaces (Hubei, China). The compounds without commercial standard were quantified using calibration curves of closely related authentic standards: rosmarinic acid for quantification of rosmarinic acid hexoside and methyl rosmarinate, salvianolic acid F for quantification of salvianolic F isomer, salvianolic acid A for quantification of salvianolic acid K, and caffeic acid for quantification of caffeoyl-threonic acid isomers. The results were expressed as µg/g of dry weight (DW).

### 2.6. Statistical Analysis

The results are presented as means ± standard error. All experiments were repeated three times. The significance of differences between lines were determined by analysis of variance (ANOVA), followed by the post hoc Tukey’s test at *p* < 0.05. The statistical analysis was carried out with Statistica 13.1 PL for Windows (StatSoft Inc., Krakow, Poland).

## 3. Results and Discussion

### 3.1. Shoot Organogenesis and Callus Induction

Previous reports on the in vitro propagation of *Salvia* species have usually focused on regeneration from apical buds of nodal explants. However, due to the strong apical dominance of this genus, it is common for clonal reproduction methods to demonstrate low multiplication ratios [13,14]. On the other hand, protocols for the effective regeneration of some species, such as *S. miltiorrhiza* or *S. nemorosa* by organogenesis, have also been described [15,16]. Therefore, the present study aims to find an effective method of regeneration of *S. bulleyana* shoots using the organogenic potential of the plant. Leaves were used as explants in the experiment, as a dozen, or even several dozen, of them can be obtained from one shoot grown in vitro; with an optimized method, such production makes it possible to obtain significant amounts of plant material in subsequent passages.

Leaves from aseptically-grown shoots of *S. bulleyana* were used as explant and cultivated on MS media with 0.1 or 0.5 mg/L NAA and one with four different cytokinins (BAP, mT, TDZ, CPPU) in three different concentrations. The response in the form of callus and/or bud formation on the explants was evaluated after 5 weeks of culture. The changes observed on explants were related to the combination of growth regulators (their type and concentration) used in the medium; the findings were consistent with previous observations described for many plants [17,18] that the regenerative capacity of the plant cells in vitro can be promoted and changed by the choice of cytokinin and its concentration [19].

**Figure 1:**
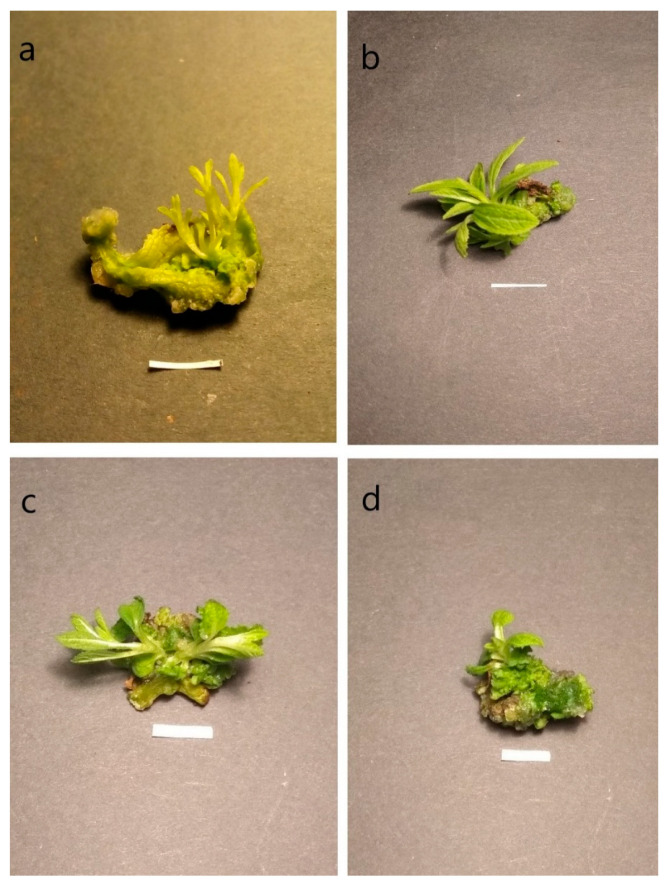
Regeneration of *S. bulleyana* through indirect organogenesis on MS medium with 0.1 mg/L NAA and 2 mg/L BAP (**a**), 0.1 mg/L NAA and 2 mg/L mT (**b**) and direct organogenesis on MS medium with 0.1 mg/L NAA and 0.2 mg/L TDZ (**c**) and 0.1 mg/L NAA and 4 mg/L CPPU (**d**) after 5 weeks. Bar 1 cm.

Plant hormones cytokinins are divided into two main types: purine-type cytokinins and phenylurea-type cytokinins. The present experiment used both types. BAP and its hydroxyl derivative mT, from the first group, showed similar response in our study, by inducing buds to develop directly from the cells of the leaves, without an intermediate stage of callus (Figure 1). In this case, considering the shoot initiation percentage and the mean bud/shoot number per explants, the best results were achieved in the medium supplemented with 0.1 mg/L NAA and 2 mg/L BAP (94.6% and 5.2, respectively) (Table 2). Under these conditions, the callus was formed only on a few explants (5.5%) from their cut edges. It was small, hard, green and showed no ability to regenerate shoots. The optimal concentration of mT for *S. bulleyana* shoot regeneration was found to be 2 mg/L in the presence of 0.1 mg/L NAA, although the results obtained were slightly weaker than in case of BAP, 76% and 3.4, respectively. When the concentrations of both mT and BAP further increased, the regeneration efficiency decreased (Table 2). Additionally, the number of differentiated buds decreased to 2.7 for BAP and 2.4 per explant for mT at a concentration of 4 mg/L. On the other hand, the frequency of callus formation on explants increased (up to almost 50% in the case of 4 mg/L mT, and 25% for 4 mg/L BAP) as the concentration of purine derivative increased. In all these cases the callus grew poorly and did not show any organogenic features. This finding is consistent with those of Fracaro and Echeverrigaray [20], who observed that lower concentrations of cytokinins favored shoot organogenesis, while higher concentrations induced callus formation in *Cunila galioides*. Ashraf et al. [21] also recorded a similar pattern for *Chlorophytum borivilianum* bud differentiation and suggested that the higher concentration of cytokinin had an inhibitory effect on shoot bud formation and elongation. As in the present results, BAP was previously the most effective for increasing the frequency and number of shoot induction on *S. miltiorrhiza* leaf explants [22]. In that study, more adventitious shoots were obtained at 1 mg/L BAP than at 2 mg/L. Shriram et al. [23] suggested that low concentrations of exogenous cytokinins (such as BAP) can effectively trigger shoot regeneration via cell differentiation stimulated by endogenous auxins.

The increase in NAA concentration to 0.5 mg/L in the presence of purine derivative cytokinins resulted in a decrease in both the frequency of adventitious bud formation and the number of buds formed on the explant, and an increase in the formation of callus tissue. Callus tissue on explants grown on these media appeared with the frequency of 60–75%. In addition, at higher cytokinin concentrations, the obtained callus sometimes covered more of the explant than just the incision sites; however, this callus tissue did not develop shoots within 5 weeks, with some explants becoming brown and necrotic at the end of this period. The combination of low NAA concentrations with cytokinin in the medium was also more efficient than higher concentrations for the formation of adventitious shoots of *Metabriggsia ovalifolia* [24]. The equilibrium between cytokinins and auxins plays a key role in promoting the meristem growth and creating new shoots. The levels of endogenous auxins, i.e., those manufactured by the apical meristem, are usually sufficient for this interaction. The supplementation of exogenous auxin may sometimes additionally support this effect. However, the observed increase in the number of shoots can be attributed to both the activity of cytokinins in stimulating bud formation and the limitation of apical dominance. Therefore, there is no need for high concentrations of auxin, which promote apical dominance, and can limit regeneration. For example, high auxin levels were inhibitory for inducing caulogenesis from mulberry tissue [25].

On the other hand, callus formation was observed on most explants of *S. bulleyana* incubated on media with urea derivative cytokinins such as TDZ and CPPU (86–100%), regardless of the auxin concentration in the medium (Table 2, Figure 1). The callus covered most of or even the entire explant (apart from the 0.5 and 1 mg/L TDZ cultures in the presence of 0.5 mg/L NAA) and caulogenesis was observed. Although shoot regeneration was noted at all tested concentrations of urea-derivative cytokinins, its frequency was significantly lower (8 to 28%) than in the presence of purine-type cytokinins. The highest frequency was reported for callus grown on medium supplemented with 0.1 mg/L NAA and 0.2 mg/L TDZ, with the mean numbers of buds/shoots per explant ranging from 2.4 to 5.2 for TDZ, and from 1.6 to 2.8 for CPPU (Table 2). In addition, in the case of TDZ increasing auxin concentration in the medium to 0.5 mg/L decreased the frequency of organogenesis and bud formation (as was for BAP and mT); the opposite effect was observed for CPPU.

Several reports suggest that TDZ was far more effective in inducing adventitious buds than other cytokinins [26,27,28]. TDZ not only demonstrates cytokinin-like activity by supporting the induction of indirect shoot organogenesis, but also it is effective in callus induction: a characteristic of auxins. Hutchinson et al. [29] reported that TDZ alone could increase endogenous auxin levels, while Capelle et al. [30] found TDZ to directly promote growth due to its own biological activities, similar to those of cytokinins, while also potentially inducing the synthesis and accumulation of endogenous cytokinins. In our study, TDZ was revealed to be more effective than CPPU for regenerating callus shoots. However, a much higher frequency of organogenesis, albeit without the involvement of callus tissue, was recorded on *S. bulleyana* leaves in the case of BAP.

### 3.2. Morphological Characterization of Obtained Shoot Lines and Their Proliferation and Growth Potential

To be successful in large scale commercial multiplication, cost-effective technology is required. Further subculturing of obtained shoot lines can significantly increase shoot induction. Therefore, the present study examined the effect of various cytokinins in the medium of line origin for the subsequent development and growth of the obtained shoots. The four well-grown shoot lines of *S. bulleyana* initiated on media with different cytokinins (1—with 2 mg/L BAP, 2—with 2 mg/L mT, 3—with 0.2 mg/L TDZ, and 4—with 4 mg/L CPPU) were transferred to MS medium with 0.1 mg/L IAA and 1 mg/L BAP. This cytokinin has been effective in in vitro cultivation of other *Salvia* species [31,32]. The lines were cultured in these conditions for further year (passage 10).

Only small variations in culture growth and morphology were found between individual lines, which in most cases, were not statistically different (Table 3). The longest main shoots (2.2 cm) and adventitious shoots (1.02 cm) were observed for the line obtained in the presence of mT. The largest number of shoots per explant (*n* = 3) was found for the same line; however, no significant discrepancies in proliferation ratio, which ranged between 2.7 to 3 shoots per explant, and only slight differences in biomass growth (0.96–1.19 FW, and 0.1–0.12 DW) were observed between lines.

**Figure 2:**
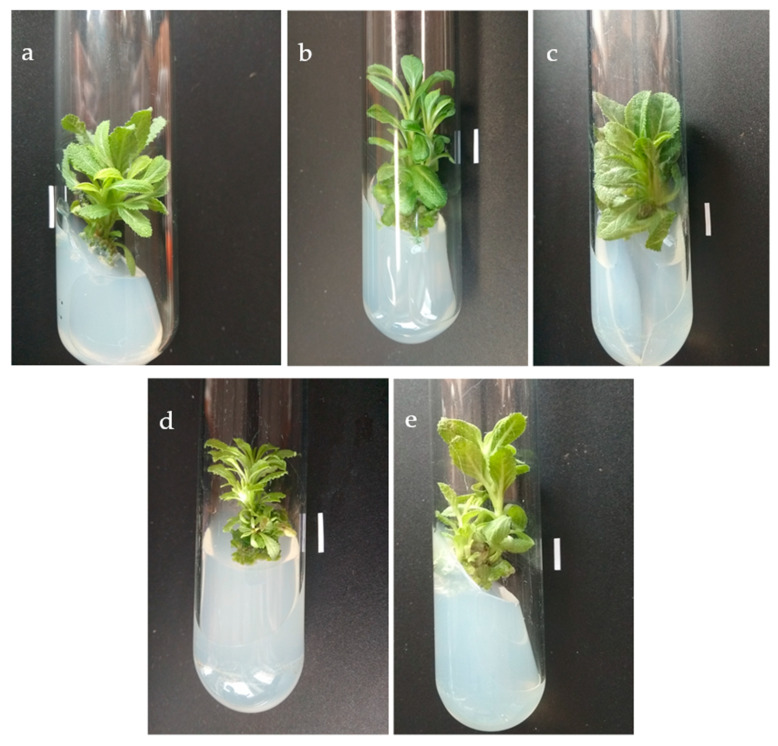
Regenerated shoot line obtained in the presence of different cytokinin: 2 mg/L BAP (**a**), 2 mg/L mT (**b**), 0.2 mg/L TDZ (**c**) and 4 mg/L CPPU (**d**), and control shoot culture (**e**) of *S. bulleyana* after 5 weeks on MS medium with 0.1 mg/L IAA and 1 mg/L BAP (passage 10). Bar 1 cm.

In most cases, within 5 weeks, the explants formed twice more buds than shoots (structures which reached at least 0.5 cm length over 5 weeks). However, in the CPPU line, six times as many buds as shoots were observed (Table 3) indicating that in this case may make it possible to obtain less material capable of subsequent micropropagation without the need to further stimulate bud elongation. Slight variations in the physical appearance of plants concerned mostly leaf shape and shoot branching (Figure 2). For instance, the plants developed via TDZ-induced organogenesis displayed a limited number of thick, wide leaves. In comparison, the leaves from CPPU-induced regenerants were slimmer and developed from small shoot branches; in general, the CPPU plants looked more “bushy”, while the control or BAP-originated plants could be described as a middle form.

### 3.3. Analysis of Genetic Stability by ISSR

Genotypic differences have been recorded in a wide range of species during shoot organogenesis [33,34]. Meanwhile, the genetic fidelity of the regenerated plants has immense practical utility and commercial implications. Therefore, the control shoots and the culture regenerants obtained via different cytokinin-induced organogenesis were screened for genetic variation based on ISSR markers. The ISSR-PCR technique was selected because of its simplicity and cost-effectiveness. It is also the method of choice to analyze organisms whose genomes have not been sequenced yet, since ISSRs are widely distributed throughout the genomic DNA, thus yielding large numbers of fragments per primer and resulting in clearer genetic inferences [35].

**Figure 3:**
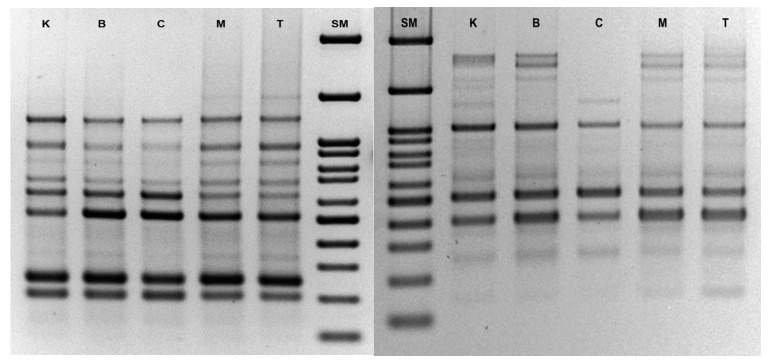
ISSR profile of control shoot culture and shoot lines obtained in the presence of different cytokinins of *S. bulleyana* (primer UBC 1—left, UBC 841—right; SM—Fast gene ruler 100 bp, K—control, B—BAP, C—CPPU, M—mT, T—TDZ).

Here, 15 microsatellite primers were used to test the genetic fidelity of *Salvia bulleyana*, based on the band reproducibly. The screening generated 664 scorable bands in total, derived from fragments of DNA ranging in length from ca. 100 to 3000 bp. When analyzing shoots from particular cytokinin cultures, the analysis based on all ISSR primers produced 137 bands for BAP, 138 bands for mT, 120 bands for CPPU, 137 bands for TDZ and 132 bands for control plants. The number of bands generated by each primer varied from two (UBC 862) to 16 (UBC 808). The ISSR profile generally revealed an acceptable level of polymorphism induction, mainly showing monomorphic banding patterns (Figure 3). The values of stability of genetic homogeneity, confirmed by the mean Jaccard coefficients of similarity, was as follows: 0.96 for BAP, 0.94 for TDZ, 0.94 for mT and 0.86 for CPPU-induced regenerants. This suggests that practically all the tested regeneration procedures provide the true-to-type *S. bulleyana* cultures and the in vitro raised shoots are free from significant somaclonal variations.

Such subtle changes in genetic fidelity are observed even in field crops of the same species [36,37]. Hence, molecular markers can often identify genetic diversity between cultivars, since slight differences in breeding environmental conditions may favor such a phenomenon. Somaclonal variation in tissue cultures is believed to be caused *inter alia* by differences in nutritional conditions, culture duration and alterations in phytohormone concentrations and auxin–cytokinin ratio. Regarding the ISSR results, it is worth noting that in vitro cultures are also considered to experience high levels of oxidative stress, which could induce variation by affecting genomic DNA and causing microsatellite instability, resulting in potential polymorphisms [38]. Indeed, the genetic instability produced under in vitro conditions has been associated with various changes at the genetic level, such as single-nucleotide changes and alterations in DNA methylation patterns [39].

Regarding the suitability of the tested regeneration protocols, the obtained level of variation in genetic fidelity is fully acceptable, as confirmed by the other parameters characterizing tissue cultures; no significant differences were observed in multiplication ratio or dry weight level, among others. Cultures induced in other species via indirect organogenesis demonstrated similar or even lower genetic similarity, as calculated based on the ISSR analysis [33,34]. In the present study, the highest variation was observed in plants developed from CPPU-induced organogenesis. This may be due to bud differentiation with the intervening callus phase, which is more vulnerable to genetic changes. Since CPPU also turned out to be the least efficient in the shoot regeneration stage, this cytokinin could be the least preferred for shoot proliferation in in vitro cultures.

Our findings support the fact that de novo propagation systems performed directly from the explant are more genetically stable than those in which regeneration occurs via the callus phase. However, callus regeneration does not have to generate somaclonal changes, as was the case with the line obtained on the medium supplemented with TDZ. The results revealed that the regenerated shoots maintained high or very high uniformity, indicating high genetic stability among the clones. Our results corroborate the reports of genetic stability obtained from existing meristems of *S. sclarea* cultures [40].

### 3.4. Polyphenol Profiling and Their Accumulation in Different Culture Lines

The phytochemical profile of the hydromethanolic extracts from four in vitro derived shoot lines and control shoots were investigated using HPLC. The compounds were identified based on their fragmentation pathway against standard compounds or the literature data [8]. Our results show that the in vitro propagated plants possess chemical profiles similar to each other and to the mother culture, confirming that no somatic mutation occurred during the in vitro propagation process (Table 4). Eleven phenolic acids and their derivatives were identified in the analyzed extracts. Seven of these, including caffeic acid, three caffeoyl-threonic acid isomers, rosmarinic acid, rosmarinic acid hexoside and salvianolic acid K, were previously reported in the methanolic extract from aerial parts of field growing *S. bulleyana* [8]. Three more, viz. methyl rosmarinate and two salvianolic acid F isomers, were detected in the roots of soil-grown plants. Detailed fragmentation data for individual compounds are presented in our previous paper [8].

All the obtained plant materials, except that generated in the presence of CPPU, was characterized by similar total polyphenol content (Table 4). The dominant compound in all analyzed extracts was rosmarinic acid (RA). It is a plant polyphenol used in many areas such as medicine, cosmetics and food supplements [41]. Many in vitro and in vivo studies from the last two decades indicate that this compound has anti-inflammatory, antioxidant, antimicrobial, hepatoprotective, neuroprotective and chemopreventive effects.

The content of RA in the extracts from the investigated shoot lines was different depending on the line and ranged from 8.1 and 15.4 mg/g DW, indicating that even the least productive shoots (obtained by organogenesis from TDZ) produced one-third RA more than shoots of plants grown in soil in the second year of vegetation [8]. It is known that the presence of growth regulators, especially cytokinins, in the growth medium affects secondary metabolite production. However, since all shoot cultures were grown for at least several months on the same medium, any observed changes in production are most likely related to differences in their metabolic potential. Such discrepancies might result from small genetic differences caused by somaclonal changes occurring at the stage of their regeneration. These reports agree with those of an earlier study reporting changes in bioactive compound production for various lines obtained during organogenesis [42].

The highest level of the dominant rosmarinic acid and also polyphenols in total was found in *S. bulleyana* shoots obtained by indirect callus organogenesis in the presence of CPPU. This is the shoot line that genetically differed the most from the mother line and other organogenetic lines, which may suggest that somaclonal changes translated into some changes in its biosynthetic pathways. Another compound whose level significantly changed in shoot cultures, and was highest in the CPPU-obtained shoot line, was salvianolic acid K (SAK). SAK content ranged in analyzed extracts from 160 to 960 µg/g DW, and the highest level detected in shoot culture was higher than found in aerial parts of field-grown plants (760 µg/g DW).

In conclusion, the lines obtained by organogenesis, which showed little genetic variability based on the ISSR analysis, demonstrated similar production profile to the mother shoots. This indicates that the regeneration procedures developed in this way guarantee that cultures are very similar to the parent plant. However, some differences in secondary metabolite accumulation were noted in the case of the most genetic varied lineage that formed on the callus in the presence of CPPU as a cytokinin. Although the close similarity to the line of origin is generally preferred in the regeneration process, the change that took place in this case turned out to be beneficial: it did not lead to significant variations in the culture growth or its phytochemical orientation, but increased the biosynthesis of valuable compounds.

## 4. Conclusions

The findings of the present study are of importance for the large-scale propagation of *S. bulleyana* for commercial purposes and for conserving the germplasm of this rare Chinese medicinal plant. The highest rate (95%) of shoot induction and the most significant number of shoots (*n* = 5.2) were achieved on MS medium containing small concentrations of NAA and BAP as cytokinin; hence this method is the most recommended approach for the effective regeneration of sage shoots. Moreover, all the tested regeneration procedures provided true-to-type *S. bulleyana* cultures, demonstrating the high genetic stability of the species and indicating its suitability for in vitro propagation methods. Still, some somaclonal changes are possible during the regeneration protocol and are worth observing, because they may result in the acquisition of new cultivars characterized by advantageous features. Such was the case of the culture obtained in the presence of CPPU that achieved higher productivity and might be further improved, e.g., for more desirable regeneration behavior.

## Figures and Tables

**Table 1:** Primers used in ISSR-PCR analysis of *S. bulleyana* shoot lines.

Primer Code	Primer Sequence 5′ → 3′	Annealing Temperature	Number of Band for Line (Cytokinin in the Medium of Origin)				
			Control	BAP	mT	TDZ	CPPU
UBC835	AGAGAGAGAGAGAGAGYC *	50 °C	10	11	11	11	9
UBC841	GAGAGAGAGAGAGAGAYC *	50 °C	13	13	12	12	9
ISSR-X1	AGAGAGAGAGAGAGAG	45 °C	6	6	6	6	6
UBC2	GAGAGAGAGAGAGAGAT	45 °C	8	8	8	8	6
UBC814	CTCTCTCTCTCTCTCTA	45 °C	10	10	11	11	10
UBC812	GAGAGAGAGAGAGAGAA	45 °C	7	7	7	7	7
ISSR-X2	CTCCTCCTCCTCRC *	45 °C	5	5	5	5	4
UBC1	ACACACACACACACACT	45 °C	11	11	11	11	10
UBC862	AGCAGCAGCAGCAGCAGC	55 °C	2	2	2	2	2
UBC808	AGAGAGAGAGAGAGAGC	48 °C	16	16	16	16	14
UBC809	AGAGAGAGAGAGAGAGG	48 °C	9	9	10	10	8
UBC840	GAGAGAGAGAGAGAGAYT *	48 °C	9	9	9	9	9
UBC 864	ATGATGATGATGATGATG	45 °C	7	10	10	10	8
UBC 818	CACACACACACACACAG	48 °C	8	8	8	8	8
UBC 834	AGAGAGAGAGAGAGAGYT *	48 °C	11	12	12	11	10

* Y = C or T; R = A or G.

**Table 2:** Effect of growth regulator combination on shoot organogenesis of *S. bulleyana* from leaf explant.

Growth Regulators [mg/L]		Callus Formation Frequency [%]	Callus Morphology	Shoot Regeneration Frequency [%]	Mean Number of Buds/Shoots Per Explant	Bud/Shoot Ratio
NAA	Cytokinin					
0.1	BAP 1	4.5	green, hard, +	77.3	5.3 ± 0.7 ^a^ (D)	78:22
	2	5.4	green, hard, +	94.6	5.2 ± 0.9 ^a^ (D)	80:20
	4	25	greenish, hard, +	66.7	2.7 ± 0.3 ^c^ (D)	86:14
0.5	1	68.8	green or greenish-white, hard, +	40.6	2.4 ± 0.4 ^cd^ (D)	87:13
	2	64.3	bright green, hard, +/++	21.4	3.3 ± 2.4 ^abc^ (D)	75:25
	4	60	green, +/++	26.7	3.0 ± 1.0 ^bcd^ (D)	100:0
0.1	mT 1	32.1	greenish, hard, +	71.4	2.7 ± 0.4 ^c^ (D)	74:26
	2	28	greenish, hard, +	76.0	3.4 ± 0.4 ^ab^ (D)	77:23
	4	48.6	green, hard, lumpy, +	68.4	2.4 ± 0.5 ^cd^ (D)	79:18
0.5	1	64.3	green, hard, +	17.9	1.8 ± 0.2 ^d^ (D)	89:11
	2	75	green, hard, +	42.9	3.3 ± 0.4 ^b^ (D)	78:22
	4	75	green, hard, +/++	38.9	3.6 ± 1.7 ^abcd^ (D)	86:14
0.1	TDZ 0.2	100	green, hard, ++/+++	27.8	3.3 ± 0.7 ^abc^ (I)	82:18
	0.5	100	greenish-white, hard, ++/+++	19.4	2.4 ± 0.7 ^cd^ (I)	94:6
	1	100	green, hard, ++	13.9	5.2 ± 1.4 ^a^ (I)	96:4
0.5	0.2	100	green, hard, ++/+++	11.1	4.5 ± 0.5 ^ab^ (I)	100:0
	0.5	100	greenish-white, hard, +/++	8.3	2.6 ± 1.5 ^bcd^ (I)	95:5
	1	88.9	green, hard, +/++	8.3	2.7 ± 0.3 ^c^ (I)	10:0
0.1	CPPU 1	86.3	green, ++	13.3	1.8 ± 0.3 ^d^ (I)	100:0
	2	100	green, soft, +++	16.7	1.6 ± 0.2 ^d^ (I)	88:12
	4	100	green, hard, ++	20.7	2.8 ± 0.5 ^c^ (I)	82:18
0.5	1	92.9	green, hard, ++	25.0	2.4 ± 0.3 ^cd^ (I)	100:0
	2	100	green, hard, ++	23.1	2.7 ± 0.5 ^c^ (I)	100:0
	4	100	green, hard, ++	18.5	2.2 ± 0.4 ^cd^ (I)	100:0

+—small callus, usually only at the incision sites, ++—the callus covers the greater part of the explant, about 50%, +++—the callus grows intensively and covers all or almost all of the explant; D—direct organogenesis, I—indirect organogenesis. Different letters after values for buds/shoot number indicates statistical differences between samples.

**Table 3:** Evaluation of morphogenic potential of S. bulleyana shoot lines obtained in the presence of different cytokinin and control shoot culture after year (passage 10).

Growth Parameter	Cytokinin in the Medium of Origin				
	BAP	mT	TDZ	CPPU	Control
Shoot forming buds/shoots [%]	84	83	80	81	85
Main shoot length [cm]	1.78 ± 0.11 ^ab^	2.19 ± 0.11 ^a^	1.74 ± 0.13 ^ab^	1.70 ± 0.09 ^b^	1.82 ± 0.12 ^ab^
Multiplication ratio	2.76 ± 0.28 ^a^	3.00 ± 0.25 ^a^	2.8 ± 0.34 ^a^	2.7 ± 0.25 ^a^	2.68 ± 0.29 ^a^
Buds/shoots ratio	76:24	67:33	68:32	86:14	64:36
Adventitious shoot length [cm]	0.88 ± 0.10 ^ab^	1.02 ± 0.11 ^a^	0.76 ± 0.08 ^b^	0.75 ± 0.10 ^b^	0.85 ± 0.08 ^ab^
Fresh weight [g]	1.04 ± 0.07 ^ab^	1.19 ± 0.07 ^a^	1.09 ± 0.06 ^ab^	0.96 ± 0.07 ^b^	1.07 ± 0.07 ^ab^
Dry weight [g]	0.11 ± 0.009 ^a^	0.12 ± 0.005 ^a^	0.11 ± 0.006 ^a^	0.10 ± 0.008 ^a^	0.11 ± 0.007 ^a^

The results are mean values ± SE. Different letters after values for the same parameter indicates statistical differences between samples.

**Table 4:** Quantitative analysis of polyphenolic compound [µg/g DW] in in vitro regenerated shoot line obtained in the presence of different cytokinin and control shoot culture of *S. bulleyana* after year (passage 10).

Compound	[M–H]	Cytokinin in the Medium of Origin				
		BAP	mT	TDZ	CPPU	Control
Caffeoyl-threonic acid I	297	56.3 ± 6.5 ^ab^	47.9 ± 0.5 ^b^	59.4 ± 0.2 ^a^	34.5 ± 0.3 ^c^	tr.
Caffeoyl-threonic acid II	297	228.8 ± 25.3 ^a^	214.5 ± 5.0 ^a^	202.7 ± 8.7 ^a^	209.3 ± 0.7 ^a^	26.6 ± 0.3 ^b^
Caffeic acid	179	341.1 ± 37.3 ^b^	174.4 ± 10.9 ^d^	432.9 ± 10.9 ^a^	173.0 ± 1.7 ^d^	211.4 ± 2.2 ^c^
Caffeoyl-threonic acid III	297	122.6 ± 11.1 ^a^	89.2 ± 1.4 ^b^	98.3 ± 2.9 ^ab^	81.1 ± 0.5 ^c^	47.4 ± 0.3 ^d^
Rosmarinic acid hexoside	521	67.0 ± 7.1 ^a^	49.5 ± 7.9 ^ab^	40.6 ± 2.6 ^b^	34.6 ± 0.3 ^b^	20.7 ± 0.4 ^d^
Rosmarinic acid	359	8991.3 ± 7776 ^b^	9142.1 ± 225.5 ^b^	8057.5 ± 281.8 ^b^	15,437.3 ± 87.6 ^a^	8829.9 ± 52.1 ^b^
Salvianolic acid K	555	163.8 ± 12.2 ^d^	272 ± 66.5 ^c^	160.2 ± 10.3 ^d^	956.7 ± 16.3 ^a^	434.6 ± 6.7 ^b^
Methyl rosmarinate	373	92.8 ± 10.8 ^c^	180.4 ± 17.1 ^b^	115.8 ± 2.0 ^c^	412.4 ± 2.8 ^a^	191.4 ± 0.3 ^b^
Salvianolic acid F isomer I	313	451.5 ± 38.9 ^b^	136.8 ± 7.3 ^d^	567.6 ± 21.4 ^a^	269.2 ± 5.5 ^c^	241.4 ± 2.9 ^c^
Salvianolic acid F isomer II	313	364.8 ± 32.7 ^a^	105.7 ± 3.6 ^c^	348.0 ± 18.1 ^a^	318.6 ± 1.1 ^a^	191.0 ± 4.5 ^b^
Total	-	10,880.2 ± 959.4 ^b^	10,412.9 ± 293.3 ^b^	10,083.0 ± 350.9 ^b^	17,926.7 ± 105.1 ^a^	10,194.4 ± 43.9 ^b^

The results are mean values ± SE. Different letters after values for the same metabolite indicates statistical differences between samples.

## Data Availability

The data presented in this study are contained within the article.
